# Phenomenology and management of cognitive and behavioral disorders in Parkinson's disease. Rise and logic of dementia in Parkinson's disease

**DOI:** 10.1186/1744-859X-5-12

**Published:** 2006-08-08

**Authors:** Constantin Potagas, Sokratis Papageorgiou

**Affiliations:** 1 Department of Neurology, University of Athens Medical School, Eginition Hospital, Athens, Greece

## Abstract

An overview of studies on the issue of dementia in Parkinson's disease shows that, over time, there has been an evolution in the perception of the magnitude of the problem and of its nature. Dementia seems today to be part of the disease. This change in the understanding of the disease can be accounted for by various methodological problems and by difficulties, on one hand, in the definition of dementia and its differentiation from other conditions, and, on the other hand, in the diagnosis of the disease itself in individual cases. Optimal therapeutic strategies are also examined, either based on cholinesterase inhibitors or antiparkinsonian drugs and symptomatic measures.

## Background: the evolution of numbers

Speaking today about cognitive and behavioral disorders in Parkinson's disease (PD) means more and more speaking about dementia. This was not the case in the beginning, when James Parkinson, in his "*Essay on the shaking palsy*" of 1817 [1], gave his well-known definition of the disease and excluded cognitive impairment: "Involuntary tremulous motion, with lessened muscular power, in parts not in action and even when supported; with a propensity to bend the trunk forward, and to pass from a walking to a running pace: *the senses and intellects being uninjured*". But, James Parkinson described just 6 patients, one of them seen from a distance; he did not have the benefit of statistics! Other early writers also denied the existence of cognitive decline. Charcot, Vulpian, Gowers thought that the intellect remained unaffected till the late stages of the disease, though Erb recognised that there were some exceptions in this rule [2].

And neither was this the case just 20 years ago, when Brown and Marsden, in 1984, in their review of the research over the 60 years prior to 1984, found a number they judged inflated (35.1%, 1 in 3 patients with PD will be demented) [3]. They adjusted these figures to a more conservative estimate of one in five patients. They proposed an estimate of the rate of dementia in PD at the range of 15% to 20%, a risk some 10% to 15% higher than the expected risk of dementia in the general population. It is true that dementia is difficult to define, identify, and understand in terms of our knowledge of the functioning of the nervous system [4]. The study of dementia in Parkinson's disease reflects this difficulty. A striking feature of the literature is the increase in the number of papers on this subject in the last 20 years (and, also, the rise of the numbers themselves in the papers).

Four years later, in a similar review of 27 studies (4,336 patients), Cummings (1988) found an average prevalence of dementia of 39.9% [5]. He noticed that studies reporting the highest incidence of intellectual impairment (69.9%) used psychological assessment techniques, whereas studies identifying the lowest prevalence of dementia (30.2%) depended on non-standardized clinical examinations. The same year (1988), Mayeux *et al *[6] in a retrospective study of records, using the DSM-IIIR, found a poor 10.9%, Girotti *et al *[7] found a little more, 14.28%. Hietanen and Teravainen [8] found that age at onset was quite an important factor as only 2% of patients with onset under 60 years were demented, in contrast to a 25% of patients with onset over 60 years.

At the beginning of the 90s Mayeux *et al *[9] reconsidered their results, and they also found a striking relation with age: 0% prevalence of dementia before 50 years and 69% in patients above age 80 years (which gives a sum prevalence of 41%) [10]. But, at this time this result was an exception. In 1995, Marder *et al *[11] found a less than two-fold risk (1.7) for PD patients to develop dementia compared to controls. In other studies of this period as well, prevalence numbers remained quite low, though rising: 18% for Pillon *et al *[12], 17.6% for Tison *et al *[13], 27.7% for Aarsland *et al *[14]. Again, Reid *et al *in 1996 [15], compared with age and made a follow-up 5 years later: they found an initial prevalence of 9% under 70 years, that was 17% at the 5-year follow-up, and 37% in patients older than 70 years, that increased to 62% after 5 years.

But, in 1999, Hobson and Meara [16] used the CAMCOG to assess intellectual impairment and found a 41% prevalence of dementia in PD patients. The less than two-fold relative risk of Marder *et al *in 1995 [11], increased to a six-fold risk (5.9) in 2001 by Aarsland *et al *[17]. And, in 2003, prevalence and incidence were found to be above 75% [18] (*See *Table 1).

**Table 1:** Evolution of numbers of dementia in Parkinson's disease

**Authors**	**Year**	**Frequency of dementia**	**Criteria – Comments**
Mayeux et al	1988	10.9%	Retrospective, DSM-III
Girotti et al	1988	14.28%	Examination, Npsy
Hietanen, Teräväinen	1988	2% < 60 yrs, 25% > 60 yrs	Examination, DSM-III
Pillon et al	1991	18%	Examination, NPsy 2SD
Mayeux et al	1992	41%, 0% < 50 yrs, 61% > 80 yrs	Examination, DSM-III
Tison et al	1995	17.6%	Examination, DSM-IIIR
Aarsland et al	1996	27.7%	Examination, DSM-IIIR
Reid et al	1996	9% < 70 yrs → 17% 37% > 70 yrs → 62%	Follow-up 5 ys
Hobson, Meara	1999	41%	Examination, CAMCOG
Marder et al	1995	1.7 Relative Risk	Follow up-controls
Aarsland et al	2001	5.9 relative risk	Follow up 4.2 ys-controls
Aarsland et al	2003	78% incidence in 8 years	Follow up 4 – 8 yrs

## Evolution of ideas about cognitive dysfunction and dementia in PD

### Cognitive deficits

Mindham judiciously called the history of dementia in PD a methodological saga [4]. Brown and Marsden, in 1984, had claimed that the reason for inflated numbers of dementia in PD were either errors in separating idiopathic Parkinson's disease from other causes of the akinetic-rigid syndrome, or errors in differentiating dementia from confusional states, depression and even ageing, or in defining and assessing dementia itself [3].

Indeed, the first reports of deterioration in intellect in PD patients appeared not long after the disease was first described [4]. However, there seemed to be a large consensus that PD patients performed significantly poorer than controls in all tests but those for language, praxis and gnosis (the "instrumental" functions), frequently showing retrieval deficits, cognitive slowing, impaired abstract thinking, and reasoning difficulties [4]. These cognitive symptoms are generally subtle and do not interfere significantly with everyday activities. However, patients and their families usually cite forgetfulness or decreased ability to follow conversations involving several persons, difficulties that are regularly attributed to a depressive state that may coexist with the disease.

If one uses appropriate neuropsychological tests, it appears that these deficits: a) are frequent, affecting up to 93% of patients according to the study by Pirozzolo et al [19]; b) they mainly affect visuospatial functioning, memory, and executive functions; and, c) they are observed even at the early stages of the disease, strongly suggesting that they are related to the subcortical pathology of the disease [20].

These selective cognitive deficits are, both phenomenologically and etiologically, somehow related to the motor syndrome or to impaired sensory-motor interaction [21].

The core deficit seems to be the "dysexecutive" syndrome. Executive functions are defined as "mental processes involved in goal-directed behavior" [22]. Patients with PD have problems in mental processes involved in the elaboration of behavioral responses to challenging situations, including the processing of relevant information, problem solving, and planning ability. Several tasks examining this require cognitive flexibility or internally guided behavior: Wisconsin Card Sorting Test, letter fluency, Stroop test, tower tasks for problem solving. All these tasks, specifically sensitive to frontal lobe lesions, are failed by patients with PD [20,23]. These failures can be associated with difficulties of patients with PD in effecting a motor plan, the higher-order control mechanism that oversees the proper operation of motor programs necessary to effect some action, such as learning to execute accurately predictable movement sequences. This same impairment in motor planning underlies the deficits in voluntary movement seen in PD patients [24].

Attention deficits are often found in patients with PD, working memory capacity is decreased, long-term memory is impaired, because of a decrease in attentional resources [23]. Especially free recall ("active memory") is impaired, whereas cued recall ("passive memory") is largely unimpaired [25]. As a whole, PD patients are impaired in tasks that involve organization of the material to be remembered, temporal ordering and conditional associative learning [20]. We must underline that PD patients are able to acquire motor or mental sets, but they learn more slowly than controls and show difficulty in maintaining newly acquired sets against competing alternatives [20,25]. Recall deficit is not primarily due to a memory disruption, since the ability to register, store, and consolidate information is preserved, but rather to difficulties in activating the neuronal processes of the functional use of memory stores. Memory scores are strongly related to performance in tests of executive functions, favoring the role of frontal lobe dysfunction in the defective activation of memory processes [20].

Olfactory impairment as found in PD patients might be considered closely related to the dysexecutive, prefrontal, syndrome; first, because of anatomical contiguity and because at least two of the Alexander basal ganglia-thalamus-frontal cortex circuits are related to structures implicated in olfaction [26]. Second, because of the behavioral observation that olfaction guides activity with yes or no reactions. PD patients seem to have a difficulty in identifying odors, not in remembering them or discriminating between them [27]. If olfaction is commonly impaired in PD, in depression and in various frontal conditions, this could be again the result of the impairment of sensory-motor interaction. Olfactory problems seem not to exist in essential tremor or in some familial forms of parkinsonism [28,29]. This, however, does not really resolve the dementia problem, because the same olfactory problems seem to be found in Alzheimer's disease (AD) and in dementia with Lewy bodies (DLB) [30,31].

The data suggest that the prominent visuospatial dysfunction results from a decrease in central processing resources rather than from a specific visuospatial dysfunction in PD patients. In any case, patients with PD have visuospatial deficits beyond their motor abnormalities (line orientation, figure assembly) [23,25].

In contrast, a more global impairment is far less common. How do we come from the above cognitive deficits to the idea of dementia?

## Generalizing cognitive deficits

### From "bradyphrenia" or slowness to dementia

There has been a debate on whether Parkinsonism results in slowness of information processing, often called bradyphrenia. Naville, in 1922, used this term to describe the chronic loss of initiative and intellectual activity that followed the acute stages of encephalitis lethargica [25]. From early on, there was a debate about the validity of bradyphrenia as a particular manifestation of PD. Because patients look "slowed down" and have lost their physical agility, observers may be tempted to generalize from the motor disabilities to cognition, and describe this as slowed, laborious and inflexible. Cognitive slowing must be differentiated from depressive and motor slowing, both of which are frequently present in PD. Anyway, slowing of information processing has frequently been reported but not consistently found. Several authors concluded that bradyphrenia is not demonstrated in PD [32]. Cognitive slowing has been clearly demonstrated only on tests that require a high level of processing, which may indicate a disturbance in cognitive strategy caused by impaired executive functioning rather than a true slowing of central processing [25].

The place of dementia in the syndrome has remained controversial because full dementia in a PD patient always makes one doubtful of the diagnosis [21], and one of the problems of the categorization of parkinsonian dementia is the neuropathological overlap with Alzheimer's disease (AD) in some post-mortem brains. Hence a categorical clinical diagnosis of parkinsonian dementia is rarely possible.

Also, the recognition of dementia in the individual subject rests on the use of criteria derived from unsatisfactory definitions and procedures. Assessments such as the WAIS bring a degree of objectivity to assessment of cognitive function; nevertheless, their use in determining the presence of dementia still involves judgment on the part of the assessors [4].

The neuropsychological picture of parkinsonian dementia that has emerged partially resembles the pattern of subcortical dementia, consisting of a dysexecutive syndrome, in the absence of genuine amnesia or impairment of instrumental activities, as with aphasia, apraxia or agnosia [33].

But, is subcortical dementia a dementia? The term, *subcortical dementia *dates back at least to the work of Kinnier Wilson in 1912, when he contrasted the cognitive failures seen in senile dementia with those in patients with subcortical lesions. He observed that these patients, though showing signs of mental deterioration, did not exhibit the amnesia, apraxia, or agnosia typical of senile dementia [25]. Authors who analyzed subcortical dementia in the 1970s speculated that the intrinsic deficit underlying subcortical dementia would be a reduction in arousal, and the cardinal element of the pattern of impairment resulting is slowness of cognition [25,33]. According to Albert *et al *subcortical dementia is characterized by four primary signs: forgetfulness, slowed cognition, personality change, poor calculating and abstracting ability [33]. However, they noted that if patients are given enough time, they can respond correctly, demonstrating that elementary verbal and perceptual capabilities are untouched by the disease.

The proposition of a "subcortical" dementia carried a degree of conviction because, indeed, the impairment seen in many subjects appeared different from that seen in AD. With time, however, many issues arose about the nature of subcortical dementia and much has been written, ranging from full acceptance of subcortical dementia as a separate syndrome to skepticism about its validity as a discrete entity [4]. Some of these questions are the following: Is subcortical dementia a clinical or a pathological concept? Do the relevant lesions reside in the subcortical region alone? Is it distinctly different from global dementias, or does the presence of extrapyramidal clinical features simply give the intellectual impairment a different character? Is subcortical dementia a stable condition or is it a prelude to global dementia? [4]

Cummings, in 1988, suggested that, in PD, dementia assumes three forms [5]: 1) a relatively mild form meeting the criteria for subcortical dementia, 2) a more severe form showing a wider and severer form of cognitive impairment but which is neuropathologically distinct from senile dementia of the Alzheimer type, and 3) a severe form of dementia showing neuropathological changes in the basal ganglia and in the cortex, the latter of the Alzheimer type. This proposal fits clinical syndromes much more satisfactorily [4]. Many neuropathological data seem also to support it.

Indeed, if specific cognitive disorders of non-demented PD patients are thought to result from subcortical lesions, many clinicopathological studies suggest the existence of three types of pathology possibly causing cognitive impairment in PD: Lewy-body-type degeneration in cortical and limbic structures, coincident AD-type pathology in cortical and limbic structures, and pathology in subcortical structures (eg, degeneration of the medial susbtantia nigra and nuclei of other ascending pathways). This leaves the door open to two opposite options: Some authors suggest that cortical or limbic Lewy body type degeneration is the main cause of dementia in PD. In many studies Lewy-body densities in the temporal or frontal cortex correlated significantly with cognitive impairment in patients with PD, independent of or in addition to AD-type pathology [34]. Others conclude that dementia in PD results from the coexistence of AD. In favor of this hypothesis, there is a high frequency of Alzheimer-like changes in the cerebral cortex, a high level of abnormal tau protein in temporal and prefrontal cortices, and marked hypoperfusion in single proton emission-computed tomography (SPECT) studies in posterior cortical regions of demented PD patients. Moreover, recently, in 200 consecutive autopsy examinations of patients with PD, 33% had moderate to severe dementia during life: 94% had cortical neuropathological changes of AD and only 3% with neuropathological changes representative of PD alone were demented [35,36]. However, some cases of dementia have been reported in PD in the absence of apparent cortical lesions that might explain the cognitive impairment [34,36], suggesting that subcortical lesions may be sufficiently severe to cause overt dementia, at least in some patients. The subcortical lesions may be themselves responsible for the frontal-like dysfunction and the inefficient activation of memory processes observed in PD patients, even in those who are demented.

Finally, the issue is not closed as significant correlations are found between neocortical Lewy-body counts and senile plaques as well as neurofibrillary tangles, which suggests common origins for these pathologies or that one triggers another.

However, we should not forget that the cognitive pattern in demented PD patients is markedly different from that of patients with AD with respect to mnemonic deficits, the intensity of the dysexecutive syndrome, and the absence of true aphasia, apraxia, or agnosia [33]. This is counterbalanced by studies that indicate that the so-called "cortical" functions are also affected in PD, albeit to a lesser extent (use of CAMCOG) [16].

It is probable that some of the cortical functions such as orientation, attention, and perception become affected later in the course of the disease. The dementia at baseline may have features of a subcortical dementia but, subsequently, aphasia, apraxia and agnosia emerge, making the dementia indistinguishable from that of Alzheimer's disease [15].

From a clinical point of view, we could suggest that dementia in PD is some final clinical image in which a non-specific dementia of either type is added to specific neuropsychological deficits of the subcortical type, already existing in PD patients. Dementia in PD is probably superimposed on previous cognitive changes in non-demented patients [35,36]. As Girotti noted [7], demented PD patients have a more severe and widespread cognitive deficit but they are affected particularly in those tests that already discriminated (non-demented) Parkinsonian patients from controls.

## Dementia and parkinsonism

### What parkinsonism?

If however we accept that a number of Parkinson patients eventually develop signs of an organic dementia, are changes in the cognition the inevitable consequence of the disease itself? Varied populations included in the studies (different stages of parkinsonism, absence of standard definition of PD itself) may be another cause for discrepancies [36].

James Parkinson had noted that the problem was one of classification: "The disease, respecting which the present inquiry is made", *though *"of a nature highly afflictive", "has not yet obtained a place in the classification of nosologists. Some have regarded its characteristic symptoms as distinct and different diseases, and others have given its name to diseases differing essentially from it" [1].

For instance, dementia with Lewy bodies and Parkinson's disease are one or two disorders? [37-39]. This particular question has been more or less resolved in an arbitrary way, by developing guidelines [40] suggesting that the term of Dementia in PD be arbitrarily restricted to patients "who have extrapyramidal motor symptoms for at least 12 months before the appearance of cognitive deterioration". However, neuropathological distinction remains problematic and, more important, it seems that cognitive impairment would be of similar severity in the terminal stage [41]. As Litvan *et al *put it, such a debate is the logical consequence of considerable clinicopathological overlap. In spite of the clinical criteria for DLB, it is always difficult to distinguish early-stage DLB from AD and PD. Also, extrapyramidal features occur in many patients with severe AD, and dementia occurs in many PD patients. On the other hand, the fact that demented PD patients progress rapidly and often respond poorly to levodopa, suggests that they may not have idiopathic PD, since autopsy studies suggest that a rapid progression of symptoms and poor levodopa response are not features of idiopathic PD [42]. Also, because many of the clinical studies include in the criteria for PD postural instability, a symptom that occurs late in PD but early in most atypical parkinsonian disorders, misdiagnosis may be common.

### Which parkinsonian patients?

Although selective cognitive changes are found in young patients and in patients with early onset of disease, demented patients are older; they may have a later age at onset of motor manifestations, longer duration of the disease, and a more rapid progression of physical disability, than non-demented patients [6,8]. When the disease begins after age 70, dementia is over three times more frequent than when the disease begins at an earlier age and, the age-specific prevalence rate of dementia for patients older than 70 is more than twice that for younger patients [8,10]. Of all the above factors there are doubts only about the age at onset of PD [43]. Akinetic-gait rigidity profile and early hallucinations are recognized as predictive factors [44,45].

As said before, it must be noted that patients with PD who develop dementia may have other atypical elements: Symmetrical disease presentation, more severe extrapyramidal signs, higher disability and bradykinesia scores, and more impairment of gait and balance, at baseline, as well as early occurrence of autonomic failure [14,15]. Also, in some studies, demented patients more often respond poorly than non-demented patients [6] and some have only moderate response to a dopamine agonist [14].

Finally, subjects with PD with dementia have greater depressive symptomatology [11,14,16]. Depression has been found to have an impact on the cognitive performance of patients with PD [46-48]. It is not clear, however, whether depressed PD patients show a reduction in cognitive capacity because of their mood problems, or whether greater cognitive impairment causes a higher risk for depression. However the relationship between these variables operates, it is important to be aware that depression may be a factor in the poor neuropsychological test performance of some PD patients.

Therefore, dementia in PD occurs after a certain period of evolution and typically in the late-onset form of the disease. We just remind that, further in his book, James Parkinson admitted that: "as the debility increases and the influence of the will over the muscles fades away, the tremulous agitation becomes more vehement". Therefore, "at the last, constant sleepiness, with slight delirium, and other marks of extreme exhaustion, announce the wished-for release" [1]. The question would be whether dementia is a part of PD or an incidental accompaniment only to be expected in the relevant age group.

In any case, we now recognize that cognitive and behavioral symptoms are common in PD and are major determinants of patients' and caregivers' distress, often leading to institutionalization. The Movement Disorders Society decided at Rome the establishment of an MDS Task Force whose task will be to define the concept of dementia in PD, develop criteria and methods for clinical diagnosis, and produce guidance on clinical management [45].

## Management

### Diagnostic points

In any case, if we are faced with a handicap, we have to manage it independently of its name, cautiously, managing depression and drug-induced situations, examining carefully the patient for other precipitating factors common in these ages, and, finally addressing dementia itself. So, the diagnostic process in patients with PD and suspected dementia involves two steps: the diagnosis of dementia and differential diagnosis of its cause. A deficit for learning new information, the hallmark for the diagnosis of dementia, is regularly reported in demented PD patients, although it is less severe than in AD.

However, diagnosis may be difficult for several reasons: Apparent impairment in certain cognitive domains may be difficult to differentiate from motor dysfunction. It may be difficult to decide if impairment in activities of daily living – essential criterion for the diagnosis of dementia – is due to cognitive or motor dysfunction. It can be hard to judge the extent to which functional impairment is attributable to cognitive dysfunction rather than motor disability. Inability to manage one's social or financial affairs may be a reasonable point to diagnose early AD, but not in a person with PD who is unable to perform these functions because of impaired mobility or severe dysphonia.

A detailed history – in order to elucidate onset, course, profile, and chronology of cognitive and behavioral symptoms – must be complemented by neuropsychological testing to identify and differentiate deficits in certain cognitive domains.

The differential diagnosis of dementia in patients with PD includes domain-specific cognitive impairments neither extensive nor severe enough to qualify as dementia, depression, confusional states due to systemic or metabolic disorders (sodium depletion, dehydratation, fever-infection), and adverse effects of drugs.

Once dementia is diagnosed, the search for the cause includes other primary degenerative dementing disorders associated with extrapyramidalfeatures and symptomatic forms of dementia due either to intracranial pathologies, such as normal pressure hydrocephalus, cerebrovascular disease, and tumors or extracranial systemic disorders, vitamin B12 deficiency, reversible dementias due to adverse effects of drugs such as anticholinergics. It is true that seeing a patient for many years may induce some "forgetting" of systematic examination at every appointment, but it is imperative that such examination is performed and reversible causes of dementia are ruled out once the diagnosis is made.

Various drug regimens can significantly interfere with the cognitive status of the patients. For instance, medications such as antihypertensive agents may cause excessive decrease of blood pressure, and antiglaucoma treatment, or benzodiazepines may create memory or concentration problems. Anticholinergic drugs may provoke acute confusional states or a permanent cognitive decline, especially if the patient is elderly, and has memory disorders, conditions in which the risk of severe damage to the ascending cholinergic system is increased. Administration of anticholinergics to patients over 70 years old should therefore be avoided, at least when they complain of memory disorders.

In the setting of cognitive impairment, it is advisable to adapt L-dopa and, especially, dopaminergic agonists in the elderly. They may provoke hallucinations and cognitive impairment. Parkinsonism responds to dopaminergic agents; however, precipitation or aggravation of hallucinosis may occur. Levodopa is preferred over dopamine agonists due to its lower propensity to cause hallucinations and somnolence. Reduction in the dose of dopaminergic agents and of other medications may be helpful in partially improving cognitive function in some cases. The balance between improvement of motor function and preservation of cognitive abilities must be weighed, and it is important for clinicians to discuss this trade-off with patients and their families.

### L-dopa, cholinesterase inhibitors, neuroleptics

Management of dementia in PD must include the patient, the caregivers as well as the social and physical environment which must be adapted to the disabilities of the patient.

It should be kept in mind that adaptation of L-dopa itself can have some beneficial effect – not only decreasing but also, in certain cases, increasing L-dopa seems to have a limited effect on cognitive impairment in PD. Subtle improvements in planning, problem solving, perceptual organization skills, visuospatial abilities, motor sequencing have been reported [49]. There is also evidence that L-dopa improves a number of aspects of executive function and verbal fluency, as well as sentence comprehension and short-term memory [50]. Such positive effects – not to be underestimated – are probably due to non-specific actions on alertness, mood, and arousal, although some more specific effects on dopaminergic transmission may exist for some components of information processing, working memory, or internal control of attention. These beneficial effects, however, may be complicated by serious side-effects such as confusion and psychoses, mainly in demented patients, as mentioned above. The beneficial effects of L-dopa on cognition are probably not shared by the dopamine agonists as, in a recent comparison of L-DOPA and the dopamine agonist pramipexole, an impairment of executive functions, verbal fluency, and memory performance was provoked by the dopamine agonist [51].

The marked cholinergic deficits in patients with DLB and PD dementia suggest the efficacy of Acetylcholinesterase inhibitors in these diseases, possibly even more than in AD for which they were originally developed [52]. Donepezil, rivastigmine and galantamine improve cognitive impairments in DLB as well as visual hallucinations, apathy, anxiety and sleep disturbances [53-56]. These three drugs have been shown to be effective in treating the cognitive and behavioural features of PD dementia, without deteriorating the motor symptoms of this disease [57-60]. However, peripheral cholinergic stimulation may produce significant side effects in patients with Parkinson's disease, and patients need to be monitored for orthostatic hypotension and diarrhoea. It has also been noted a possible worsening of extrapyramidal symptomatology [61].

Psychotic symptoms such as hallucinations and delusions are frequently seen in demented patients with PD. Compared with conventional neuroleptics, the newer atypical antipsychotic agents may be associated with lower rates of extrapyramidal side effects. We must also be careful because of the extreme sensitivity of patients with DLB to the extrapyramidal side effects of neuroleptic medications. Traditionnal neuroleptics (D2 receptor antagonists) should be avoided as they exagerbate parkinsonism and can provoke severe hypersensitivity reactions in up to 50% of patients with high mortality [62]. Atypical antipsychotics in the treatment of psychosis associated with PD seem to be effective with an acceptable safety risk. Newer atypical neuroleptics like clozapine, olanzapine, quetiapine have weak affinity for D1 and D2 receptors and are lacking extrapyramidal side effects. They have been proven safer and efficacious for demented PD patients [63-65]. However even these drugs should be used with caution in patients with DLB and PD dementia.

Depression, which is frequent in these patients is preferably treated with SSRIs, which lack the anticholinergic side effects of tricyclics.

## Competing interests

The author(s) declare that they have no competing interests.
